# Pseudoaneurisma de aorta abdominal como complicação de pancreatite crônica: relato de caso

**DOI:** 10.1590/1677-5449.006316

**Published:** 2017

**Authors:** Eduardo Carvalho Horta Barbosa, Leonardo Pires de Sá Nóbrega, Daniel Augusto de Souza Rodrigues, Josué Rafael Ferreira Cunha, Claudio Eluan Kalume

**Affiliations:** 1 Hospital de Base do Distrito Federal – HBDF, Unidade de Cirurgia Vascular, Brasília, DF, Brasil.

**Keywords:** pseudoaneurisma, aorta abdominal, pancreatite

## Abstract

A pancreatite crônica é uma enfermidade associada a diversas complicações vasculares, como pseudocisto hemorrágico, trombose do sistema venoso portal e formações varicosas e pseudoaneurismáticas. O pseudoaneurisma de aorta abdominal secundário à pancreatite crônica é uma complicação rara, de difícil suspeição clínica, que requer tratamento complexo. A fisiopatologia dessa condição envolve a corrosão enzimática tecidual após a liberação e ativação de enzimas exócrinas proteolíticas das células acinares do pâncreas. O presente estudo relata o caso de um paciente de 52 anos, etilista crônico, internado com dor abdominal difusa, cuja propedêutica revelou se tratar de um pseudoaneurisma em aorta infrarrenal. Optou-se pelo tratamento cirúrgico convencional, levando-se em consideração a idade, as condições clínicas do paciente e a disponibilidade de endopróteses compatíveis com o diâmetro da aorta.

## INTRODUÇÃO

A pancreatite é uma condição clínica que apresenta alta incidência e prevalência em todo o mundo. Estima-se que haja, nos Estados Unidos, 56.000 internações anuais por pancreatite crônica^1^. No Brasil, segundo o Departamento de Informática do Sistema Único de Saúde (DATASUS), a incidência de pancreatite aguda é de 15,9/100.000 habitantes por ano. As complicações vasculares relacionadas à pancreatite não são comuns, ocorrendo em uma frequência que varia entre 1,2-14%^1^.

As lesões arteriais relacionadas à pancreatite acometem com maior frequência a artéria esplênica, que representa 40% dos casos. Em seguida, vêm as artérias gastroduodenal (30%), pancreaticoduodenal (20%), gástrica (5%) e hepática (2%)^2^.

O pseudoaneurisma de aorta abdominal associado à pancreatite é uma condição extremamente rara, com apenas três casos relatados na literatura^3^. Este estudo tem como finalidade relatar um caso de pseudoaneurisma de aorta abdominal secundário à pancreatite crônica, atendido em hospital terciário da rede pública e tratado por cirurgia convencional, com interposição de prótese de dácron aortoaórtica.

## RELATO DO CASO

**Figura 1:**
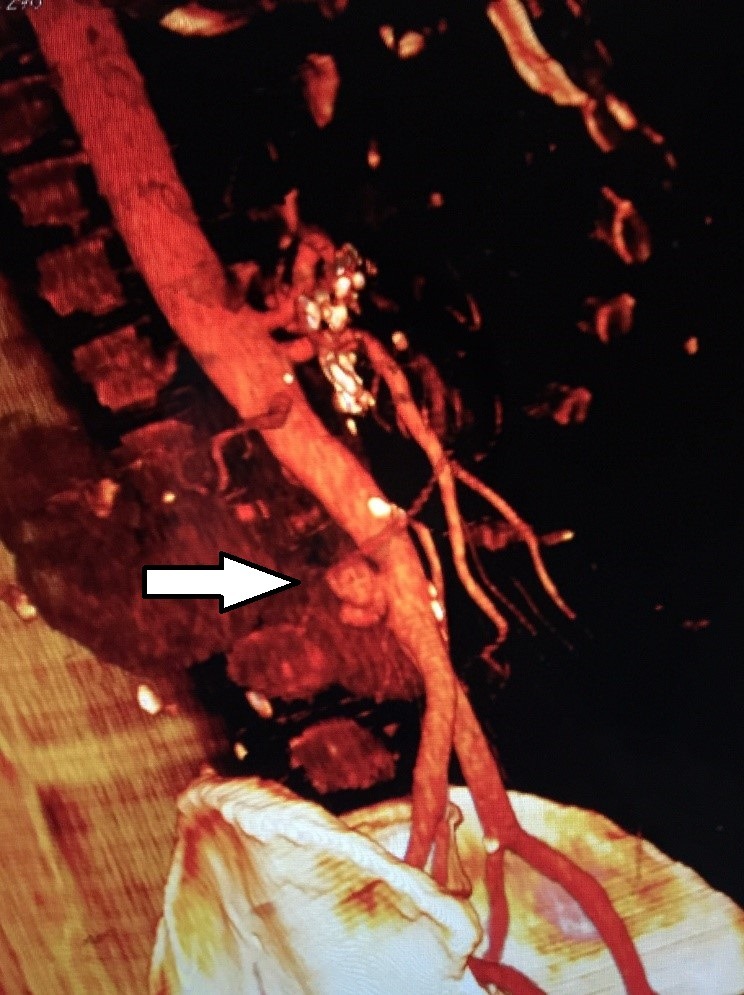
Angiotomografia de aorta abdominal evidenciando pseudoaneurisma na parede posterior.

Paciente masculino, 52 anos, hipertenso, diabético, tabagista, etilista desde os 19 anos, procurou a equipe de clínica médica do pronto-socorro com queixa de dor em barra, em abdome superior, associada a náuseas e diarreia crônica. Relatava história pregressa de diversas internações clínicas para tratamento de pancreatite crônica agudizada. Negava trauma, cirurgias prévias, intervenções endovasculares, cardiopatias ou doenças reumáticas. Uma avaliação complementar com exames laboratoriais e tomografia abdominal com contraste venoso, além de confirmar um novo episódio de pancreatite crônica agudizada, ainda evidenciou a presença de um possível aneurisma de aorta abdominal. Após estabilização clínica e melhora do quadro agudo da pancreatite, foi encaminhado para a unidade de cirurgia vascular, onde uma angiotomografia revelou a presença de um pseudoaneurisma de aorta abdominal infrarrenal, distante cinco centímetros da bifurcação das artérias ilíacas (Figura 1).

**Figura 2:**
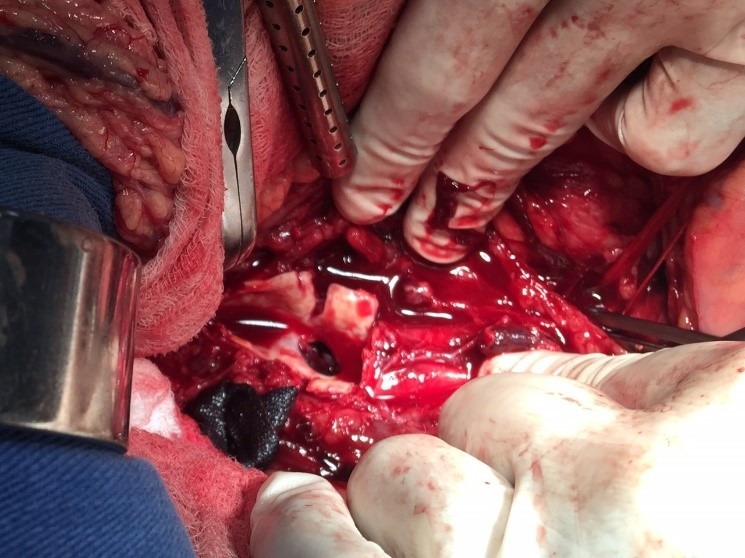
Óstio do pseudoaneurisma da aorta abdominal.

**Figura 3:**
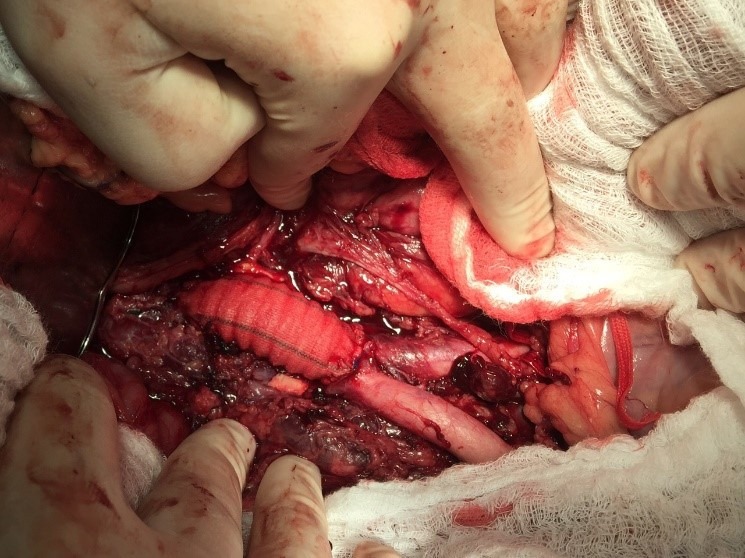
Resultado final após interposição da prótese de dácron.

Optou-se por correção convencional do pseudoaneurisma através de incisão xifopúbica e acesso transperitoneal. Após clampeamento aórtico e arteriotomia da parede anterior, foi identificado o óstio do pseudoaneurisma na parede posterior da aorta (Figura 2). Realizou-se interposição de prótese de dácron com anastomose terminoterminal aortoaórtica (Figura 3).

**Figura 4:**
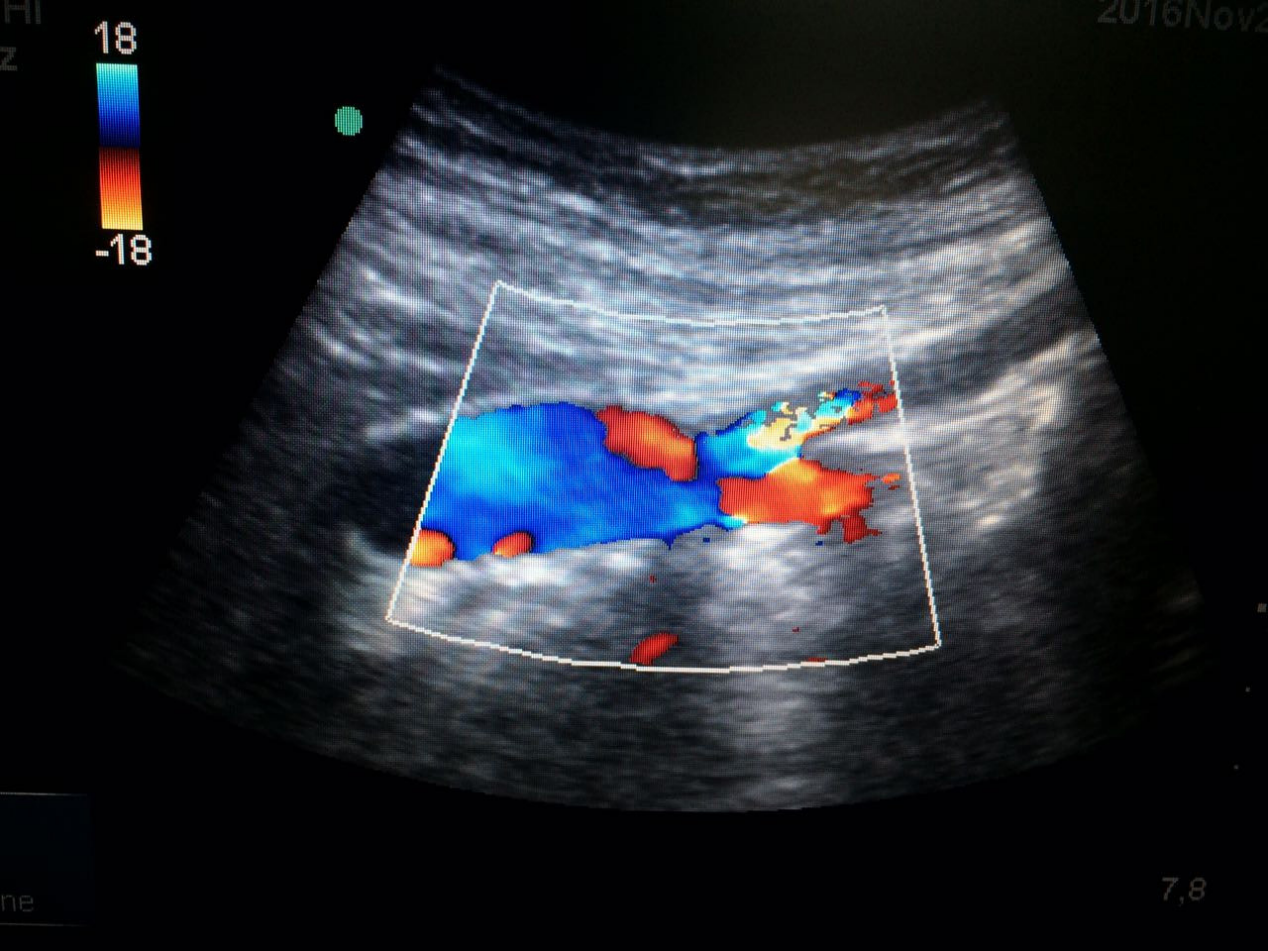
Ultrassonografia com Doppler para controle pós-operatório, realizada 6 meses após o procedimento, mostrando enxerto pérvio, sem estenoses, dilatações ou formações pseudoaneurismáticas, em corte longitudinal.

O paciente evoluiu bem no pós-operatório, recebendo alta hospitalar após cinco dias de internação. Encontra-se em acompanhamento ambulatorial mensal com avaliações periódicas do enxerto através de ultrassonografia vascular (Figura 4). Até o presente momento, evolui sem intercorrências e sem novos episódios de exacerbação da pancreatite.

## DISCUSSÃO

A pancreatite associa-se a diversas complicações vasculares, como pseudocisto hemorrágico, trombose do sistema venoso portal, formações varicosas e pseudoaneurismas^2^. Essa combinação leva a uma morbimortalidade elevada, e a sobrevivência desses pacientes depende diretamente do diagnóstico e tratamento precoce.

Durante o processo fisiopatológico inicial da pancreatite, há liberação e ativação de enzimas exócrinas proteolíticas das células acinares, como a tripsina, que podem causar lesões não restritas apenas às estruturas adjacentes ao pâncreas, podendo acometer ossos, fígado, células sanguíneas e vasos^4^. A formação de pseudoaneurismas arteriais é consequência dessa corrosão enzimática tecidual. Diversos fatores de risco contribuem para a formação dos pseudoaneurismas, incluindo pancreatite necrotizante, falência de múltiplos órgãos, coleções de fluidos pancreáticos e abscessos^1^. A evolução natural da doença é imprevisível, variando entre a regressão espontânea e a ruptura para cavidade abdominal, retroperitônio ou trato gastrointestinal. Sabe-se que o risco de ruptura não se relaciona diretamente ao tamanho da formação pseudoaneurismática^2^.

Os pacientes portadores de pseudoaneurismas secundários à pancreatite podem apresentar quadros clínicos diversos, variando de assintomáticos a casos com dor abdominal, distensão, melena, sangramento intermitente de pequena monta e até hemorragia aguda (taquicardia, hipotensão)^5^. O paciente em questão, apesar do pseudoaneurisma de aorta abdominal, manteve-se hemodinamicamente estável em toda a internação.

Os exames de imagem complementares são fundamentais para o diagnóstico do pseudoaneurisma, uma vez que grande parte dos portadores de pancreatite já apresenta dor abdominal crônica, como no caso relatado. Ademais, muitos deles possuem história de abuso de álcool e, nesses casos, os sangramentos podem ser erroneamente justificados pela presença concomitante de doença ulcerosa péptica e varizes esofágicas, comum nessas situações. Nem mesmo a hemorragia aguda severa é de fácil percepção devido ao frequente estado de falência múltipla de órgãos nesses indivíduos.

O manejo do pseudoaneurisma de aorta abdominal secundário à pancreatite depende da condição clínica do paciente e da sua estabilidade hemodinâmica^1^. No paciente estável, a ultrassonografia abdominal com Doppler geralmente é o primeiro exame diagnóstico realizado. Pode sugerir envolvimento vascular e identificar trombose venosa, áreas necróticas e abscessos cavitários, porém seus achados são inespecíficos^5^. A angiotomografia e a angiografia são métodos mais acurados que permitem o diagnóstico e a intervenção terapêutica em pacientes selecionados.

A rotina de rastreio de pseudoaneurismas secundários não está bem estabelecida na literatura. Entretanto, Suzuki et al. recomendam a realização de eco-Doppler abdominal em intervalos regulares em pacientes portadores de pancreatite crônica, como forma de prevenção secundária^6^.

Os pseudoaneurismas são mais propensos à ruptura do que aneurismas verdadeiros. Dessa forma, a indicação de tratamento cirúrgico, quer seja convencional ou endovascular, deve ser realizada tão logo seja possível^7^.

A cirurgia convencional para tratamento do pseudoaneurisma de aorta abdominal associado à pancreatite é definida como o padrão-ouro em pacientes instáveis^2^. Nos casos em que há estabilidade hemodinâmica, o tratamento ideal é controverso, devido ao pequeno número de casos relatados^3^. Em todos os relatos identificados na literatura, a opção terapêutica adotada foi a correção por técnica aberta com exclusão do pseudoaneurisma e interposição de prótese sintética.

As técnicas de reparo endovascular representam uma alternativa à cirurgia convencional^8^^,^ 9, pois eliminam os traumas cirúrgicos dos acessos trans ou retroperitoneal e do clampeamento da aorta, com possível redução dos índices de morbimortalidade associados ao procedimento. Por outro lado, a técnica aberta ainda é uma opção efetiva e segura, com resultados em longo prazo bem estabelecidos na literatura. É preferível para os pacientes jovens, com expectativa de vida elevada e que possuam reserva fisiológica compatível com laparotomia e clampeamento aórtico^10^^,^ 11. Esses fatores foram decisivos na escolha pela cirurgia convencional, em função da idade e da condição clínica do paciente. Outro fator importante considerado na escolha da técnica terapêutica foi a incompatibilidade do diâmetro da aorta infrarrenal, que media 1,6 centímetro, com a endoprótese de aorta disponível no serviço, o que requereria o uso *off-label* de uma endoprótese para extensão ilíaca.

Pode-se concluir que o pseudoaneurisma de aorta abdominal secundário à pancreatite é uma complicação rara, de difícil diagnóstico clínico e com possível evolução desastrosa. Dessa forma, é essencial que a suspeita de complicações vasculares associadas à pancreatite crônica, como ocorrido nesse caso, esteja sempre presente entre as hipóteses diagnósticas.
