# Supplementary material for: Pseudoaneurisma de aorta abdominal como complicação de pancreatite crônica: relato de caso
Source: J Vasc Bras. 2017 Jul-Sep;16(3):244–7. [Article in Portuguese] doi: 10.1590/1677-5449.006316 (PMC5868942; doi:10.1590/1677-5449.006316)
Supplement: Translation

# Translation: Abdominal aortic pseudoaneurysm as a complication of chronic pancreatitis: case report

**Authors:** Eduardo Carvalho Horta Barbosa, Leonardo Pires de Sá Nóbrega, Daniel Augusto de Souza Rodrigues, Josué Rafael Ferreira Cunha, Claudio Eluan Kalume

**Affiliations:** 1 Hospital de Base do Distrito Federal – HBDF, Unidade de Cirurgia Vascular, Brasília, DF, Brazil.

**Keywords:** pseudoaneurysm, abdominal aorta, pancreatitis

## Abstract

Chronic pancreatitis can be complicated by several vascular disorders, such as bleeding pseudocysts, thrombosis of the venous portal system, varicosities, and pseudoaneurysms. Pseudoaneurysm of the abdominal aorta secondary to chronic pancreatitis is a rare complication. It is a challenging clinical situation, demanding a high degree of clinical suspicion, and requires complex therapeutic procedures. The pathophysiology of this condition involves interstitial liberation and activation of enzymes from the exocrine pancreatic glands and subsequent digestion of the surrounding tissues. In the present case report, we describe a 52-year-old patient complaining of diffuse abdominal pains. Clinical investigation revealed chronic alcoholism and imaging examinations showed a pseudoaneurysm of the infrarenal aorta. We decided to perform conventional surgical treatment after considering the patient’s age and clinical status and the endoprostheses available at our hospital with diameters compatible with the patient’s aorta.

## INTRODUCTION

Pancreatitis is a clinical condition with high incidence and prevalence worldwide. In the United States, it is estimated that there are 56,000 hospital admissions for chronic pancreatitis every year.^1^ In Brazil, according to the Brazilian National Health Service’s IT Department (DATASUS), the incidence of acute pancreatitis is 15.9/100,000 inhabitants per year. Vascular complications related to pancreatitis are uncommon, with a frequency of occurrence that varies from 1.2 to 14%.^1^

Arterial injuries related to pancreatitis most frequently involve the splenic artery, accounting for 40% of cases, followed by, in descending order of frequency, the gastroduodenal (30%), pancreaticoduodenal (20%), gastric (5%), and hepatic arteries (2%).^2^

Abdominal aortic pseudoaneurysm associated with pancreatitis is an extremely rare condition and there are only three cases reported in the literature.^3^ This article reports a case of abdominal aortic pseudoaneurysm secondary to chronic pancreatitis, seen at a tertiary hospital on the public healthcare system and treated using conventional surgery to construct a dacron aortic interposition graft.

## CASE REPORT

A 52-year-old male patient, who was hypertensive, diabetic, and a smoker and had a history of chronic alcoholism since the age of 19, presented to the clinical team in the emergency room complaining of pain all across the upper abdomen associated with nausea and chronic diarrhea. He described a previous history of several clinical admissions to treat acute exacerbations of chronic pancreatitis. He had not been subject to trauma, previous surgery, or endovascular intervention and did not have heart disease or rheumatic disease. Workup with laboratory tests and abdominal tomography with venous contrast confirmed a new episode of acute exacerbation of chronic pancreatitis and also revealed evidence suggestive of an abdominal aortic aneurysm. After clinical stabilization and improvement of the acute pancreatitis, he was referred to the vascular surgery service, where angiotomography reveled an infrarenal abdominal aortic pseudoaneurysm, five centimeters from the bifurcation of the iliac arteries (Figure 1).

**Figure 1:** Angiotomography of the abdominal aorta, showing a pseudoaneurysm of the posterior wall.

The decision was taken to perform conventional surgery to repair the pseudoaneurysm, via a xiphopubic incision and transperitoneal access. After clamping the aorta and performing arteriotomy of the anterior wall, the pseudoaneurysm ostium was identified in the posterior wall of the aorta (Figure 2). An aorta to aorta dacron interposition graft was constructed with end-to-end anastomosis (Figure 3).

**Figure 2:** Ostium of the abdominal aortic pseudoaneurysm.

**Figure 3:** Final result after interposition of the dacron graft.

The patient recovered well during the postoperative period and was discharged after 5 days in hospital. He is currently attending monthly follow-up consultations with regular assessments of the graft using vascular ultrasonography (Figure 4). To date he has been free from intercurrent conditions and has not suffered further episodes of exacerbation of his pancreatitis.

**Figure 4:** Longitudinal ultrasonography with Doppler for postoperative control, conducted 6 months after the procedure, showing the graft patent, with no stenosis, dilatations, or pseudoaneurysm formation.

## DISCUSSION

Pancreatitis can be complicated by several vascular disorders, such as bleeding pseudocysts, thrombosis of the venous portal system, varicosities, and pseudoaneurysms.^2^ This combination results in elevated morbidity and mortality rates and among these patients survival is directly dependent on diagnosis and early treatment.

During the initial pathophysiologic process of pancreatitis, proteolytic exocrine enzymes such as trypsin are released from acinar cells and activated and these enzymes can cause damage that is not restricted to structures adjacent to the pancreas, but can involve bones, liver, and blood cells and vessels.^4^ Formation of arterial pseudoaneurysms is a consequence of enzymatic corrosion of tissues. Many different risk factors contribute to formation of pseudoaneurysms, including necrotizing pancreatitis, multiple organ failure, accumulation of pancreatic fluids, and abscesses.^1^ The natural progression of this disease is unpredictable, varying from spontaneous regression to rupture into the abdominal cavity, retroperitoneal space, or gastrointestinal tract. It is known that the risk of rupture is not directly related to pseudoaneurysm size.^2^

Patients with pseudoaneurysms secondary to pancreatitis may exhibit a variety of clinical presentations, ranging from asymptomatic cases with abdominal pains, through distension, melena, and minor intermittent bleeding, to acute hemorrhage (tachycardia, hypotension).^5^ The patient in question remained hemodynamically stable throughout his time in hospital, despite the abdominal aortic pseudoaneurysm.

Imaging exams play a fundamental role in diagnosing pseudoaneurysms, since a large proportion of patients with pancreatitis already have chronic abdominal pains, as in the case reported here. Furthermore, many of them have a history of alcohol abuse and in these cases bleeding may be erroneously attributed to concomitant peptic ulcer disease and esophageal varices, which are common in these cases. Even severe acute hemorrhage is not simple to detect because of the frequency of multiple organ failure in these patients.

Management of abdominal aortic pseudoaneurysm secondary to pancreatitis is driven by the patient’s clinical condition and hemodynamic stability.^1^ For stable patients, abdominal ultrasonography with Doppler is generally the first diagnostic examination used. It can suggest vascular involvement and identify venous thrombosis, necrotic areas, and abscesses in the abdominal cavity, but findings are nonspecific.^5^ Angiotomography and angiography are more accurate methods that enable diagnosis and therapeutic intervention in selected patients.

There is no well-established screening routine in the literature for secondary pseudoaneurysms. However, Suzuki et al. recommend abdominal Doppler ultrasonography at regular intervals in patients with chronic pancreatitis, as a form of secondary prevention.^6^

Pseudoaneurysms have a greater propensity to rupture than true aneurysms. Therefore the decision to perform surgery, whether conventional or endovascular, should be arrived at as soon as possible.^7^

In unstable patients, conventional surgery is defined as the gold standard treatment for abdominal aortic pseudoaneurysm associated with pancreatitis.^2^ In cases in which there is hemodynamic stability, the ideal treatment is controversial because of the small number of case reports that have been published.^3^ In all of the reports identified in the literature, the treatment option chosen was repair with an open technique, with exclusion of the pseudoaneurysm and interposition of a synthetic graft.

Endovascular repair techniques offer an alternative to conventional surgery,^8^^,^ 9 since they eliminate the surgical traumas of transperitoneal or retroperitoneal access and aorta clamping, with a possible reduction in the rates of morbidity and mortality associated with the procedure. On the other hand, the open technique is still a safe and effective option, with long term results that are well-established in the literature. It is preferable for younger patients, who have longer life expectancy and physiological reserves compatible with laparotomy and aortic clamping.^10^^,^ 11 These factors were decisive in our choice of conventional surgery, in view of the age and clinical condition of the patient. Another important factor considered in choice of the therapeutic technique was the incompatibility between the diameter of the patient’s infrarenal aorta, which measured 1.6 centimeters, and the aortic endoprosthesis that was available at our service, which would have obliged us to use an iliac extension endoprosthesis off label.

It can be concluded that abdominal aortic pseudoaneurysm secondary to pancreatitis is a rare complication that is difficult to diagnose clinically and involves the possibility of catastrophic progression. It is therefore essential that vascular complications associated with chronic pancreatitis are always included in the list of diagnostic hypotheses, as was the case here.
